# Transient Behavior of a Queueing Model with Hyper-Exponentially Distributed Processing Times and Finite Buffer Capacity

**DOI:** 10.3390/s22249909

**Published:** 2022-12-16

**Authors:** Wojciech M. Kempa, Iwona Paprocka

**Affiliations:** 1 Department of Mathematics Applications and Methods for Artificial Intelligence, Faculty of Applied Mathematics, Silesian University of Technology, 23 Kaszubska Str., 44-100 Gliwice, Poland; 2 Department of Engineering Processes Automation and Integrated Manufacturing Systems, Faculty of Mechanical Engineering, Silesian University of Technology, Konarskiego 18A Str., 44-100 Gliwice, Poland

**Keywords:** finite capacity, hyper-exponential distribution, queue-size distribution, transient analysis, end-of-line product, digital twin, disassembly sequencing

## Abstract

In the paper, a finite-capacity queueing model is considered in which jobs arrive according to a Poisson process and are being served according to hyper-exponential service times. A system of equations for the time-sensitive queue-size distribution is established by applying the paradigm of embedded Markov chain and total probability law. The solution of the corresponding system written for Laplace transforms is obtained via an algebraic approach in a compact form. Numerical illustration results are attached as well.

## 1. Introduction

Current companies support the circular economy strategy by adapting the life-cycle models of their products to the circular one [1]. Enterprises deal with the recovery of materials, especially valuable and limited natural resources of the Earth. The list of regulations on prevention and protection of the earth’s environment can be found in [2]. The directive also sets a minimum level for material recovery and recycling [3]. In a closed product cycle, raw materials or semi-finished products are obtained through recycling, disassembly, sorting and re-engineering. The recovered raw material or semi-finished product is transferred to producers, steel mills and foundries for further processing.

Proper balancing, line sequencing [4], as well as consideration of planning and scheduling disassembly tasks are essential for effective use of the disassembly system. In the disassembly line balancing problem, operations (tasks) are distributed evenly between the disassembly line stations, so that the idle time at the stations is as short as possible. Before balancing the disassembly line, the following must be known [5]: disassembly operation times, sequence relationships between operations and the size of the production cycle or the number of stations. The balancing result is the determination of the smallest number of stations in a given cycle time or the determination of the lowest cycle value for a given number of disassembly stations. The problem of estimating the times of disassembly operations was considered in [6]. In order to establish a sequential relationship between operations, it is necessary to predict the frequency of product arrival and its quality.

Uncertainty management is a priority in disassembly due to uncertain quality, quantity and return timing of an end-of-line product [7]. A product often breaks down at the end of its life cycle, components are missing and worn connections can be difficult to disconnect over time due to wear, rust, or deformation. Disassembly lines balancing problems are hampered by possible differences in the duration of the disassembly task (related to joint condition), as well as in the destination node representing the target component for re-manufacturing, reusing, or recycling. In the disassembly process, both the wear, quality, and return frequency of the disassembled product should be taken into account. Thanks to reliable predictions of machine occupancy and buffer saturation with three types of target component, it is possible to carry out a balance of tasks along with production capacities and to predict the start and execution times of orders. These activities are aimed at fully utilizing constrained production capacity of the disassembly system.

The problems are to design an effective disassembly line and provide an efficient approach to solve the disassembly line balancing problem with a high degree of disassembly uncertainty [8]. Due to unknown quality, quantity and frequency of a product inflow, the primary objective of this paper is to estimate the inter-arrival time of the product with the target component for re-manufacturing, reusing or recycling received from the reverse logistics network. The second objective is to compute the probability of occupancy and release of the disassembly system for given inter-arrival times, disassembly times and the size of the entering buffer. Estimating both inter-arrival times and disassembly times based on a product quality gains value, rather than deriving mean times without learning from the past.

The rest of the paper is organized as follows: the literature review is presented in Section 1.1. A model of a product disassembly sequencing line with three possible variants: re-manufacturing, reusing or recycling is presented in Section 2. The method of estimating the probability of disassembly system occupancy for three disassembly product variants is presented in Section 3, Section 4, Section 5 and Section 6. Comparison of sequences obtained by means of computer simulations for the estimated inter-arrival times and disassembly times, together with necessary analysis, is presented in Section 7. A brief summary of the results and future research objectives are presented in Section 8.

### 1.1. Literature Review

The literature on the subject identifies the problems of planning the disassembly sequence for production lines dedicated to a single product [1,9,10,11], mixed products [12,13,14], and multiple products [15,16,17]. In this paper, a single product with possible variants of disassembly for recycling, reusing, and re-engineering depending on historical information about the inter-arrival times of possible variants. The presented model can be adopted to mixed-product problems when a product variant is assigned to a disassembly system with a certain probability. Additionally, the presented model can be adopted to multi-product problems when precedence relationships of each product are presented in a combined precedence graph with the number of possible alternatives depending on a certain probability.

Disassembly tree, directed graph (network) [7], AND/OR graph [11,18,19], hierarchical tree diagram or disassembly precedence diagram can be used to describe a disassembly line sequencing problem. The advantage of the AND/OR graph is a clear presentation of disassembly relationships between joints, especially in the case of reuse or recycling [11]. In the AND/OR graph a node represents a part/sub-assembly (a disassembly task) of a product [19]. The disassembly task with the highest priority is assigned to the disassembly station under the condition that the cycle time is not exceeded. A disassembly sequence is the only decision in the selective case until the target components are achieved in the case of repairing defective components or reusing valuable components [20].

The state-based approach including multiple possible disassembly paths in an AND/OR graph for a product with different components and sub-assemblies is considered in [12]. For the stochastic disassembly balancing problem, only one general disassembly path is chosen for all product variants. An example of a single precedence graph based on different end-of-life states of a product is presented in [13]. In the precedence graph, a node represents a disassembly task for each product component. Boysen et al. [14] reduced the mixed-model balancing problem to a single-product case. Models represent all variations of the same base product that differ in specific custom product attributes. The joint precedence graph is formed based on the models of product variants using pairs of triangular XOR nodes that express that the connected options are mutually exclusive. “Xor” means that only one of the disassembly options should be selected. The estimated joint disassembly times are based on the probability of product version and task times. Different products reach the disassembly system at an average rate in [17]. The precedence relationships of each product are presented in a combined precedence graph with no model relationships to increase the number of possible alternatives. A task is assigned to a disassembly station with a certain probability. If the probability is lower than the threshold set for assigning the task to the disassembly station, the task is assigned to the corresponding station.

Usually in the field of engineering, inter-arrival times [18], connection states [19], disassembly times [13] are previously assumed. Related literature deals with uncertainty by estimating the disassembly time using correction factors for disassembly tools based on a data mining process of product geometric features [10]. In addition, the parameters that best describe the element disassembly operation are force, tools, disassembly mechanism, part repeatability, joint recognition, product structure, accessibility, positioning and basic time. The operation time is estimated by summing these values, expressed in time measurement units (TMUs) [21]. The task disassembly time can also be estimated as 10 × MU × 0.036, where 0.036 is the standard conversion value from task TMUs to seconds [22,23]. It is also assumed that disassembly times are described either by normal [13,24] and exponential [25] distributions, or observed standard average times [15,26].

The disassembly line balancing problem with fuzzy processing times is presented in [27]. Bentaha et al. [28] developed the Monte-Carlo method for modeling product task times as a random variable with a given probability distribution. The problem of disassembly sequence planning, taking into account the fuzzy quality of the element affecting the time and cost of operation, is presented in [29,30]. Rickli and Camelio [7] developed an approach to uncertainty management basing on the assumption that the age distribution of the end-of-life product is known and related to product quality. The product value curve (life-cycle value) of the end-of life product is described by a negative exponential distribution. Reveliotis [31] introduced reinforcement learning to update quality distribution information on a product quality uncertainty. Gao et al. [32] modelled uncertainty using fuzzy logic to update status of end-of-life product quality after each inspection operation.

The AND/OR graph is the most popular approach for multiple possible disassembly paths [12] different end-of-life states of a product [13] probable product version [14]. The most similar approach, where the graph is adapted to the possible variants of the product for reuse or recycling [11] and for repairing defective components or reusing is presented in [20]. The greatest advantage and the most distinguishing feature of our approach is (1) a possible product variant with a target component for regeneration, reuse or recycling according to the Poisson distribution and (2) processed in the time described by the hyper-exponential distribution obtained from historical data on disassembly times. (3) The time-sensitive queue-size distribution is established for the disassembly system by applying the paradigm of embedded Markov chain and total probability law. The compact algebraic approach enables the disassembly system to be represented in its digital twin.

### 1.2. Goals and Approaches

The highlight of this paper is listed as follows:

- The problem of sequencing a single-product line with three possible cases of a target component is investigated: recycling, reuse, or regeneration (remanufacturing). The presented method of estimating the disassembly sequence is important for the research development and results from an urgent need [33].
- A finite-capacity queueing disassembly model is considered in which jobs arrive according to a Poisson process and are being served according to hyper-exponential service times. A system of equations for the time-sensitive queue-size distribution is established by applying the paradigm of embedded Markov chain and total probability law. The solution of the corresponding system written for Laplace transforms is obtained via algebraic approach in a compact form what is important in building a digital twin of the disassembly system.
- A method of estimation inter-arrival time and disassembly time of the end-of-life product with various quality based on historical information is proposed. The objective is to maximize the throughput of the disassembly system with the predicted sequence.

## 2. Queueing Model

In the article, we consider a queueing model with the arrival stream governed by a Poisson process with rate λ and a finite capacity of an accumulating buffer. Namely, the maximum system state equals *B*, so we have a buffer with B−1 places and one place at the service station. An arriving job that finds the system in state *B* (so the buffer being saturated and the service station busy with processing) is lost and leaves the system without service,

The processing of arriving jobs is organized according to the FIFO service discipline, and the service time is hyper-exponentially distributed with the cumulative distribution function (CDF) of the form

(1) $$F\left(t\right)\overset{def}{=}\sum_{i=1}^{k}p_{i}\left(1-e^{-\mu_{i}t}\right)$$

The probability density function (PDF) is given as

(2) $$f\left(t\right)\overset{def}{=}\sum_{i=1}^{k}p_{i}\mu_{i}e^{-\mu_{i}t},$$

where t>0, $p_{i}>0,\mu_{i}>0$ (for i=1,…,k), $\sum_{i=1}^{k}p_{i}=1$ and k∈N is predefined.

In consequence, with probability $p_{i}$ the service time has exponential distribution with mean $\mu_{i}^{-1}$, where i=1,…,k.

Let us denote

(3) $$Q_{n}\left(t,m\right)\overset{def}{=}P\left\{X\left(t\right)=m\;|\;X_{0}=n\right\},$$

where X(t) stands for the number of jobs present in the system at time *t*.

In summary, in this section, a precise mathematical description of the considered queueing model was presented. In particular, the information about the arrival process of incoming jobs, the service process, and the system size was given, as well as the notation which will be used in the next sections.

## 3. Time-Dependent Equations for System Behavior

Assume that the system is empty at the opening (at time t=0).

We have the following equation:

(4) $$Q_{0}\left(t,m\right)=\lambda \int_{0}^{t}e^{-\lambda x}Q_{1}\left(t-x,m\right)dx+I\left(m=0\right)e^{-\lambda t},$$

where I(A) denotes the indicator of the random event A.

Similarly, if the system starts its evolution with *n* jobs present in the accumulating buffer, where n∈{1,…,B}, we have the following representation:

(5) $$\begin{array}{cc} Q_{n}\left(t,m\right)= & \sum_{j=0}^{B-n-1}\int_{0}^{t}Q_{n+j-1}\left(t-x\right)\frac{{\left(\lambda x\right)}^{j}}{j!}e^{-\lambda x}\sum_{i=1}^{k}p_{i}\mu_{i}e^{-\mu_{i}x}dx\\ & +\sum_{j=B-n}^{\infty }\int_{0}^{t}Q_{B-1}\left(t-x\right)\frac{{\left(\lambda x\right)}^{j}}{j!}e^{-\lambda x}\sum_{i=1}^{k}p_{i}\mu_{i}e^{-\mu_{i}x}dx+\gamma_{n}\left(t\right), \end{array}$$

where

(6) $$\begin{array}{cc} \gamma_{n}\left(t\right)\overset{def}{=} & e^{-\lambda t}\left(1-\sum_{i=1}^{k}p_{i}\left(1-e^{-\mu_{i}t}\right)\right)\\ & \cdot \left(I\left(n\leq m\leq B-1\right)\frac{{\left(\lambda t\right)}^{m-n}}{(m-n)!}+I\left(n=B\right)\sum_{j=B-n}^{\infty }\frac{{\left(\lambda t\right)}^{j}}{j!}\right). \end{array}$$

In this section, a system of integral Volterra-type equations for time-dependent (transient) queue-size distribution conditioned by the initial buffer state was established. In the case of the buffer being non-empty at the starting moment, the formula of total probability with respect to the first departure moment after t>0 was used.

## 4. Governing Equations in Terms of Laplace Transforms

Introduce Laplace transforms of conditional distributions $Q_{n}\left(t,m\right)$, n∈{0,…,B} in the following way:

(7) $$q_{n}\left(s,m\right)\overset{def}{=}\int_{0}^{\infty }e^{-st}Q_{n}\left(t,m\right)dt,\;s>0.$$

Observe that, utilizing Fubini’s theorem and changing the order of integration, we have for fixed i,j, and *r*

(8) $$\begin{array}{cc} & \int_{t=0}^{\infty }e^{-st}dt\int_{x=0}^{t}Q_{i}\left(t-x,m\right)\frac{{\left(\lambda x\right)}^{j}}{j!}e^{-\lambda x}p_{r}\mu_{r}e^{-\mu_{r}x}dx\\ & =p_{r}\mu_{r}\int_{x=0}^{\infty }e^{-(\lambda +\mu_{r}+s)x}\frac{{\left(\lambda x\right)}^{j}}{j!}dx\int_{t=x}^{\infty }e^{-s(t-x)}Q_{i}\left(t-x,m\right)dt\\ & =\frac{\lambda^{j}p_{r}\mu_{r}}{{\left(\lambda +\mu_{r}+s\right)}^{j+1}}\int_{x=0}^{\infty }\frac{{\left(\lambda +\mu_{r}+s\right)}^{j+1}}{j!}x^{j}e^{-(\lambda +\mu_{r}+s)x}dx\int_{y=0}^{\infty }e^{-sy}Q_{i}\left(y,m\right)dy\\ & =\frac{\lambda^{j}p_{r}\mu_{r}}{{\left(\lambda +\mu_{r}+s\right)}^{j+1}}q_{i}\left(s,m\right). \end{array}$$

Similarly, we get for fixed *i* and *j*

(9) $$\begin{array}{cc} & \int_{0}^{\infty }e^{-st}p_{i}\left(1-e^{-\mu_{i}t}\right)e^{-\lambda t}\frac{{\left(\lambda t\right)}^{j}}{j!}dt\\ & =p_{i}\frac{\lambda^{j}}{{\left(\lambda +s\right)}^{j+1}}\int_{0}^{\infty }\frac{{\left(\lambda +s\right)}^{j+1}}{j!}t^{j}e^{-(\lambda +s)t}dt\\ & -p_{i}\frac{\lambda^{j}}{{\left(\lambda +\mu_{i}+s\right)}^{j+1}}\int_{0}^{\infty }\frac{{\left(\lambda +\mu_{i}+s\right)}^{j+1}}{j!}t^{j}e^{-(\lambda +\mu_{i}+s)t}dt\\ & =p_{i}\lambda^{j}\left(\frac{1}{{\left(\lambda +s\right)}^{j+1}}-\frac{1}{{\left(\lambda +\mu_{i}+s\right)}^{j+1}}\right). \end{array}$$

Observe that, denoting

(10) $${\hat{\gamma }}_{n}\left(s\right)\overset{def}{=}\int_{0}^{\infty }e^{-st}\gamma_{n}\left(t\right)dt,$$

The Formula (9) leads to the following representation:

(11) $$\begin{array}{cc} & {\hat{\gamma }}_{n}\left(s\right)=\left\{\begin{array}{cc} \lambda^{m-n}\sum_{i=1}^{k}p_{i}\left(\frac{1}{{\left(\lambda +s\right)}^{m-n+1}}-\frac{1}{{\left(\lambda +\mu_{i}+s\right)}^{m-n+1}}\right),\; & \mathrm{if}\;1\leq n\leq m\leq B-1,\\ \sum_{i=1}^{k}p_{i}\sum_{j=B-n}^{\infty }\lambda^{j}\left(\frac{1}{{\left(\lambda +s\right)}^{j+1}}-\frac{1}{{\left(\lambda +\mu_{i}+s\right)}^{j+1}}\right),\; & \mathrm{if}\;n=B,\\ 0,\; & \mathrm{otherwise}\;\mathrm{for}\;n\geq 1. \end{array}\right. \end{array}$$

Applying Laplace transforms to both sides of Formulae (4) and (5) and referring to (7) and (8), we obtain the following system of equations written for Laplace transforms of conditional queue-size distribution in the considered model:

(12) $$q_{0}\left(s,m\right)=\frac{\lambda }{\lambda +s}q_{1}\left(s,m\right)+\frac{\delta_{m,0}}{\lambda +s}$$

and

(13) $$\begin{array}{cc} & q_{n}\left(s,m\right)=\sum_{j=0}^{B-n-1}\sum_{i=1}^{k}\frac{\lambda^{j}p_{i}\mu_{i}}{{\left(\lambda +\mu_{i}+s\right)}^{j+1}}q_{n+j-1}\left(s,m\right)\\ & +q_{B-1}\left(s,m\right)\sum_{j=B-n}^{\infty }\sum_{i=1}^{k}\frac{\lambda^{j}p_{i}\mu_{i}}{{\left(\lambda +\mu_{i}+s\right)}^{j+1}}+{\hat{\gamma }}_{n}\left(s\right), \end{array}$$

where n∈{1,…,B}.

Taking, additionally,

(14) $$a_{j}\left(s\right)\overset{def}{=}\sum_{i=1}^{k}\frac{\lambda^{j}p_{i}\mu_{i}}{{\left(\lambda +\mu_{i}+s\right)}^{j+1}},$$

We can simplify (13) as follows:

(15) $$q_{n}\left(s,m\right)=\sum_{j=0}^{B-n-1}a_{j}\left(s\right)q_{n+j-1}\left(s,m\right)+q_{B-1}\left(s,m\right)\sum_{j=B-n}^{\infty }a_{j}\left(s\right)+{\hat{\gamma }}_{n}\left(s\right),$$

where n∈{1,…,B}.

In summary, in this section, a system of integral equations obtained in Section 3 was rewritten as a linear system in terms of Laplace transforms. Moreover, it was simplified to a form that is more convenient for using the matrix notation.

## 5. Matrix Form and the Solution

Let us supplement the definition of the functional sequence $\left({\hat{\gamma }}_{n}\left(s\right)\right)$ (see (10)) by taking

(16) $${\hat{\gamma }}_{0}\left(s\right)\overset{def}{=}\frac{\delta_{m,0}}{\lambda +s}.$$

Next, introduce (B×B)-size functional matrix $\tilde{A}\left(s\right)=\left({\tilde{a}}_{i,j}\left(s\right)\right)$ in the following way:

(17) $$\begin{array}{cc} & {\tilde{a}}_{i,i}\left(s\right)=\left\{\begin{array}{cc} 1,\; & i=0,\\ 1-a_{1}\left(s\right),\; & 1\leq i\leq B-1,\\ 1-\sum_{j=1}^{\infty }a_{j}\left(s\right),\; & i=B,\\ 1\; & i=B+1, \end{array}\right. \end{array}$$

For 1≤i≤

(18) $$\begin{array}{cc} & {\tilde{a}}_{i,j}\left(s\right)=\left\{\begin{array}{cc} -a_{0}\left(s\right),\; & 1\leq i\leq B,\;j=i-1,\\ -\sum_{j=0}^{\infty }a_{j}\left(s\right),\; & i=B+1,\;j=B,\\ -a_{j-i+1}\left(s\right),\; & 0\leq i+1\leq j\leq B-1,\\ -\sum_{r=B-i}^{\infty }a_{r}\left(s\right),\; & 1\leq i\leq B,\;j=B,\\ 0,\; & \mathrm{otherwise}, \end{array}\right. \end{array}$$

So

(19) $$\begin{array}{cc} & \tilde{A}\left(s\right)\overset{def}{=}\left[\begin{array}{cccccccc} 1 & -\frac{\lambda }{\lambda +s} & 0 & 0 & \ldots & 0 & 0 & 0\\ -a_{0}\left(s\right) & 1-a_{1}\left(s\right) & -a_{2}\left(s\right) & -a_{3}\left(s\right) & \ldots & -a_{B-2}\left(s\right) & -\sum_{B-1}^{\infty }a_{j}\left(s\right) & 0\\ 0 & -a_{0}\left(s\right) & 1-a_{1}\left(s\right) & -a_{2}\left(s\right) & \ldots & -a_{B-3}\left(s\right) & -\sum_{B-2}^{\infty }a_{j}\left(s\right) & 0\\ 0 & 0 & -a_{0}\left(s\right) & 1-a_{1}\left(s\right) & \ldots & -a_{B-4}\left(s\right) & -\sum_{B-3}^{\infty }a_{j}\left(s\right) & 0\\ \vdots & \vdots & \vdots & \vdots & \ddots & \vdots & \vdots & \vdots \\ 0 & 0 & 0 & 0 & \ldots & -a_{0}\left(s\right) & 1-\sum_{j=1}^{\infty }a_{j}\left(s\right) & 0\\ 0 & 0 & 0 & 0 & \ldots & 0 & -\sum_{j=0}^{\infty }a_{j}\left(s\right) & 1 \end{array}\right]. \end{array}$$

Putting now

(20) $$Q\left(s\right)\overset{def}{=}{\left[q_{0}\left(s\right),\ldots ,q_{B}\left(s\right)\right]}^{T}$$

and

(21) $$\Gamma \left(s\right)\overset{def}{=}{\left[{\hat{\gamma }}_{0}\left(s\right),\ldots ,{\hat{\gamma }}_{B}\left(s\right)\right]}^{T},$$

We can rewrite the Equations (12) and (15) as a matrix-form system as follows:

(22) $$\tilde{A}\left(s\right)Q\left(s\right)=\Gamma \left(s\right).$$

Due to the fact that (22) is a Cramer’s system and has exactly one solution given by the formula

(23) $$Q\left(s\right)={\left(\tilde{A}\left(s\right)\right)}^{-1}\Gamma \left(s\right).$$

In this section, a system of linear equations found for Laplace transforms of conditional queue-size distribution was written applying matrix notation. In addition, a formula for a general-type solution of the system was given.

## 6. Special Case-A System without Accumulating Buffer

A special case of the considered queuing model is a system without accumulating buffer in which *B* = 1. In this case we have

(24) $$\tilde{A}\left(s\right)=\left[\begin{array}{cc} 1 & -\frac{\lambda }{\lambda +s}\\ -\sum_{j=0}^{\infty }a_{j}\left(s\right) & 1 \end{array}\right]$$

and

(25) $$Q\left(s\right)\overset{def}{=}{\left[q_{0}\left(s\right),q_{1}\left(s\right)\right]}^{T},\;\Gamma \left(s\right)\overset{def}{=}{\left[{\hat{\gamma }}_{0}\left(s\right),{\hat{\gamma }}_{1}\left(s\right)\right]}^{T}.$$

Because

(26) $$\det\tilde{A}\left(s\right)=\frac{1}{1-\frac{\lambda }{\lambda +s}\sum_{j=0}^{\infty }a_{j}\left(s\right)},$$

The solution can be written explicitly by the following formula:

(27) $$\tilde{Q}\left(s\right)=\frac{1}{1-\frac{\lambda }{\lambda +s}\sum_{j=0}^{\infty }a_{j}\left(s\right)}\left[\begin{array}{cc} 1 & \frac{\lambda }{\lambda +s}\\ \sum_{j=0}^{\infty }a_{j}\left(s\right) & 1 \end{array}\right]\left[\begin{array}{c} \frac{\delta_{m,0}}{\lambda +s}\\ {\hat{\gamma }}_{1}\left(s\right) \end{array}\right].$$

In summary, in this section, a special case of the considered queueing model was studied, in that there is no possibility of waiting for incoming jobs (no buffer). In this case it is possible to write the solution in the explicit form and the appropriate formula was given.

## 7. Numerical Study

In this section, we investigate numerically the impact of main “input” parameters of the disassembly system (such as arrival intensity, disassembly rate, traffic load for successive scenarios for different quality of disassembled products, or mean vacation duration) on the queue-size distribution. Matlab is used to obtain the transient queue-size distribution from the Formula (25) for individual system parameters. In the we are dealing with a model described as follows:

- Poisson arrivals of products with rate λ;
- CDF of the disassembly time of arriving jobs is a mixture of three exponential distributions (one for regeneration, reuse or recycling) and is defined as (1) with mean $\mu_{1}^{-1}$, $\mu_{2}^{-1}$, $\mu_{3}^{-1}$ and given probability for disassembly variant (quality) $p_{1}$, $p_{2}$, $p_{3}$.

A hyper-exponential distribution of disassembly time is used to model the type of service: regeneration, reuse or recycling, for which the average disassembly times vary significantly. The value of parameter $p_{i}$ indicates the version/quality of an upcoming task, which takes an average of $\mu_{i}^{-1}$ time units.

### 7.1. Impact of Buffer State

**Figure 1:**
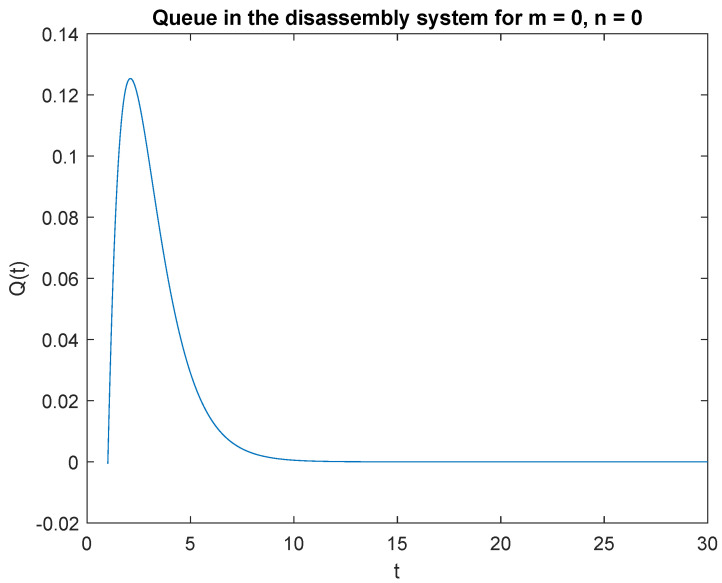
Impact of buffer state on probability P{X(t)=m=0|X(0)=n=0}.

First, let us investigate the effect of the initial state B of the input buffer on the transient queue-size distribution. Consider the scenario where λ=1, which corresponds to the rate of arrival of a product of unknown quality every 60 s: regeneration, reuse, or recycling. The DCF parameters of the disassembly time is $\mu_{1}=4$, $\mu_{2}=3$, and $\mu_{3}=2$, which gives the average disassembly speed of 15, 20, and 30 s, respectively, for regeneration, reuse, or recycling. The traffic load values for the successive scenarios are 20%, 30%, and 50% for regeneration, reuse, or recycling, respectively. Taking $p_{i}=\left\{0.2,0.3,0.5\right\}$ and a special case of the queueing model where capacity of the accumulating buffer B=1, in Figure 1 probabilities that we have no job, m=0 in the disassembly system at the time *t* and at the beginning there is no job, n=0 in the input buffer, $P\{X\left(t\right)=0\;|\;X_{0}=0\}$ are presented.

**Figure 2:**
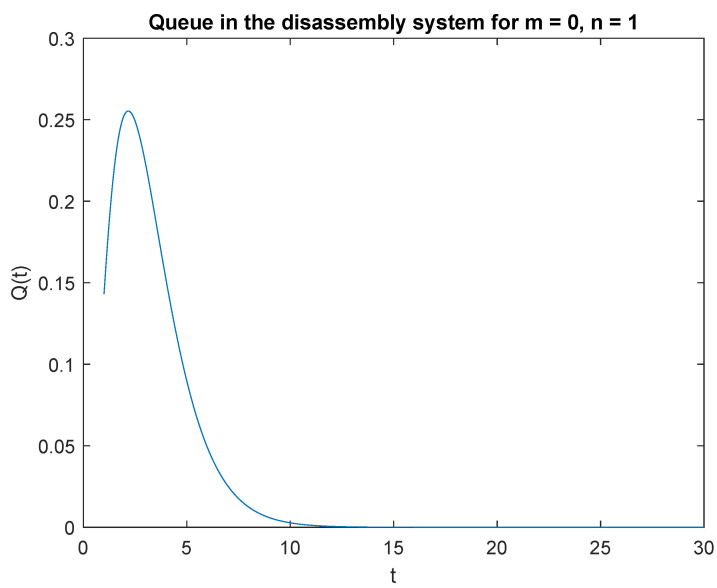
Impact of buffer state on probability P{X(t)=m=0|X(0)=n=1}.

Observe that the probability $P\{X\left(t\right)=0\;|\;X_{0}=0\}$ increases shortly after opening the disassembly system to 2.2 s (Figure 1). After reaching the peak, the probability of no job after 2.2 s decreases. Similarly, the probability $P\{X\left(t\right)=0\;|\;X_{0}=1\}$ is greatest in the 2.3 s of the simulation (Figure 2). The probability of no job drops to zero after 14 s of the simulation.

**Figure 3:**
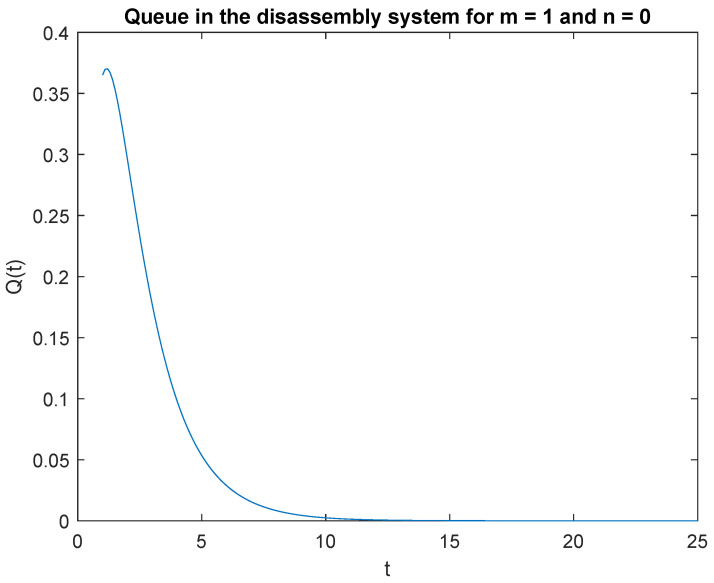
Impact of buffer state on probability P{X(t)=1|X(0)=0}.

**Figure 4:**
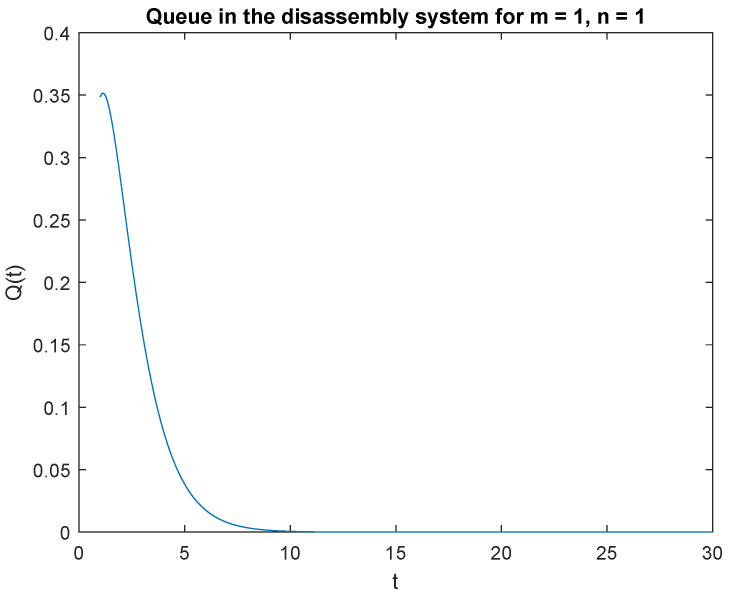
Impact of buffer state on probability P{X(t)=1|X(0)=1}.

Note that the probability $P\{X\left(t\right)=1\;|\;X_{0}=0\}$ is highest right after the system is opened (Figure 3). This is due to the fact that the system starts disassembly as soon as it is opened. The disassembly system immediately goes into the vacation mode due to lack of disassembly tasks. After 2.2 s, the probability of a job, $P\{X\left(t\right)=1\;|\;X_{0}=1\}$ drops to zero after 14 seconds of simulation (Figure 4).

Summarizing the considered probabilities for two cases: at the beginning there is no job *n* = 0 or is only one job *n* = 1 in the input buffer, we can conclude that: the probability of one job, *m* = 1 in the disassembly system at time *t* is greater than the probability of no job, *m* = 0 at time *t* for both cases *n* = 0 and *n* = 1.

### 7.2. Impact of Buffer Capacity

**Figure 5:**
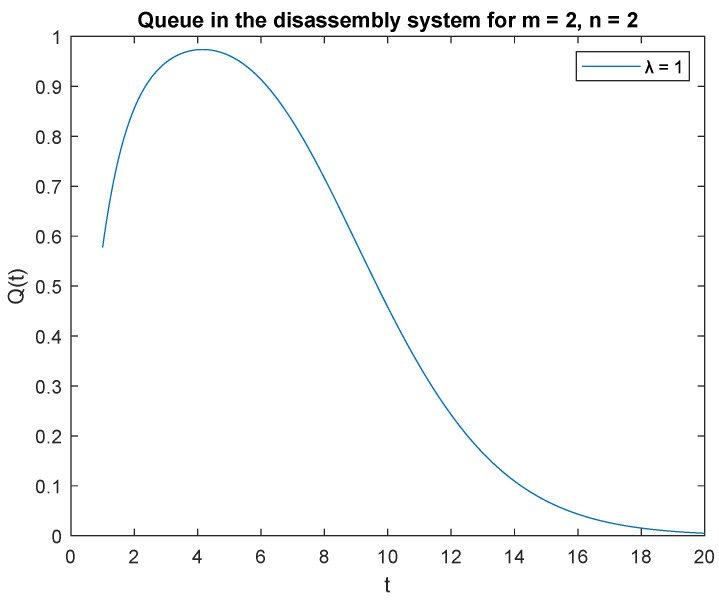
Impact of buffer capacity on probability P{X(t)=2|X(0)=2}.

**Figure 6:**
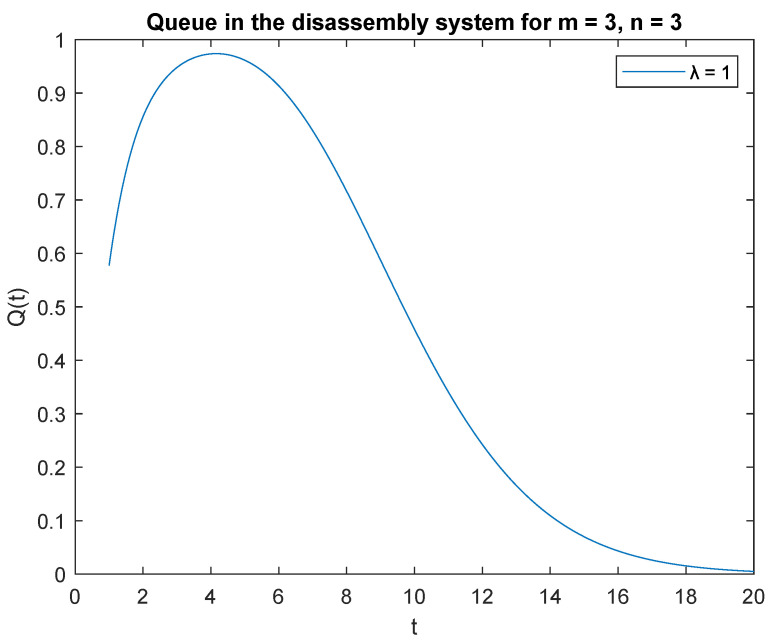
Impact of buffer capacity on probability P{X(t)=3|X(0)=3}.

Now observe the transient behavior of the considered probabilities, where capacity of the accumulating buffer *B* = 2. Average disassembling and traffic load rates for subsequent scenarios remain the same for regeneration, reuse, or recycling as in previous simulations. In Figure 5, probabilities of two jobs, *m* = 2 in the system at time *t* and two jobs, *n* = 2 in the input buffer at the beginning, *PX*(*t*) = 2|*X*(0) = 2 are presented. The probability *PX*(*t*) = 2 | *X*(0) = 2 increases as soon as the system is opened (Figure 5) and reaches its highest value after 4 s of the disassembly process. After 20 s of simulation, the disassembly system goes into the vacation mode. The same phenomenon is noticeable for probability *PX*(*t*) = 3 | *X*(0) = 3 where capacity of the accumulation buffer *B* = 3 (Figure 6).

**Figure 7:**
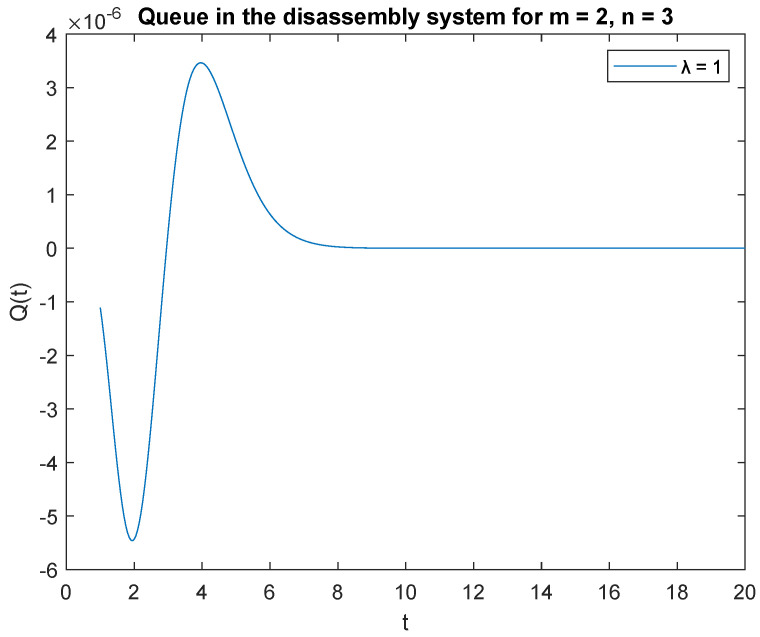
Impact of buffer capacity on probability P{X(t)=2|X(0)=3}.

In the case of probability *PX*(*t*) = 2 | *X*(0) = 3 and the capacity of the accumulating buffer *B* = 3, the transient behavior of the considered probabilities goes into the vacation mode much earlier than in the previous cases (Figure 7). Once the peak is reached, the probability of having jobs drops to zero within 8 s. Summarizing the effect of buffer capacity, the busy time of the disassembly system increases with capacity. Knowing the time of transition to vacation mode is crucial to balancing tasks with production capacity.

### 7.3. Impact of Arrival Rate

**Figure 8:**
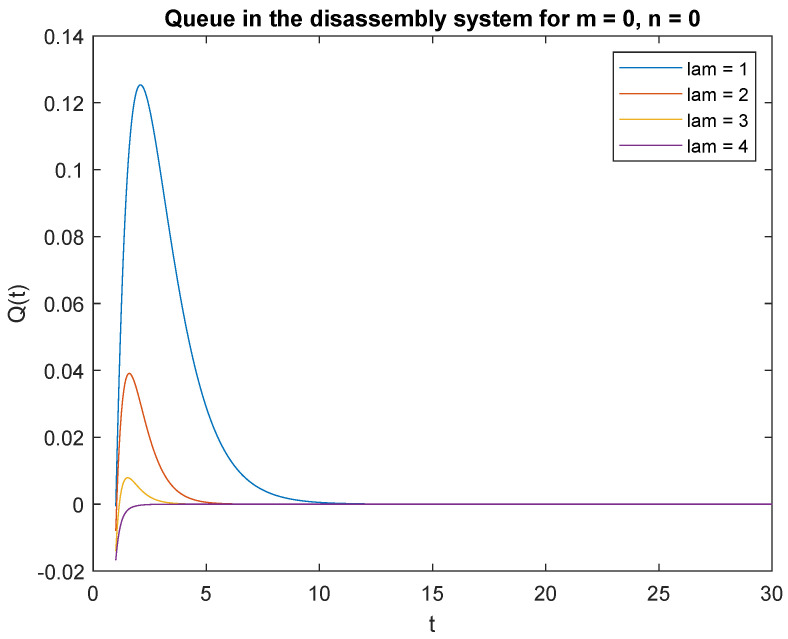
Impact of arrival rate on probability P{X(t)=m=0|X(0)=n=0}.

Let us investigate the impact of the arrival rate λ on the transient queue-size distribution. Consider four scenarios where the inter-arrival rate described by λ=1,2,3, which corresponds to inter-arrival rate of 60, 30, 20, and 30 for a product. The CDF parameter describing the disassembly time is $\mu_{1}=4$, $\mu_{2}=3$ and $\mu_{3}=2$ for regeneration, reuse, and recycling, respectively. The traffic load value is 20%, 30%, and 50% for regeneration, reuse, and recycling, respectively. Considering the special case of the queuing model where capacity of the accumulating buffer *B* = 1, Figure 8 presents the probabilities of no job, *m* = 0 in the disassembly system at time *t* and of no job *n* = 0 in the input buffer at the beginning, $P\{X\left(t\right)=0\;|\;X_{0}=0\}$ with changing arrival rates for products with unknown quality.

**Figure 9:**
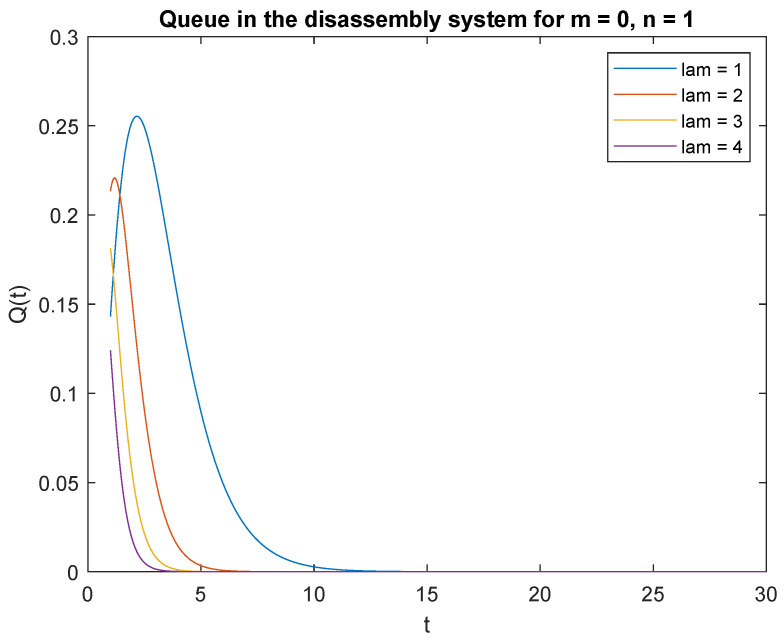
Impact of arrival rate on probability P{X(t)=m=0|X(0)=n=1}.

Observe probabilities $P\{X\left(t\right)=0\;|\;X_{0}=0\}$ (Figure 8) and $P\{X\left(t\right)=0\;|\;X_{0}=1\}$ (Figure 9), the transient conditional distributions increase as inter-arrival rates decrease λ=1, λ=2, λ=3, and lambda=4. The transient conditional distributions increase shortly after the opening of the disassembly system for inter-arrival rates λ=1, λ=2, (Figure 8 and Figure 9). The transient conditional distributions decrease constantly after opening the disassembly system for inter-arrival rates λ=3, λ=2, starting from the peak at the beginning of the simulation (Figure 8 and Figure 9).

**Figure 10:**
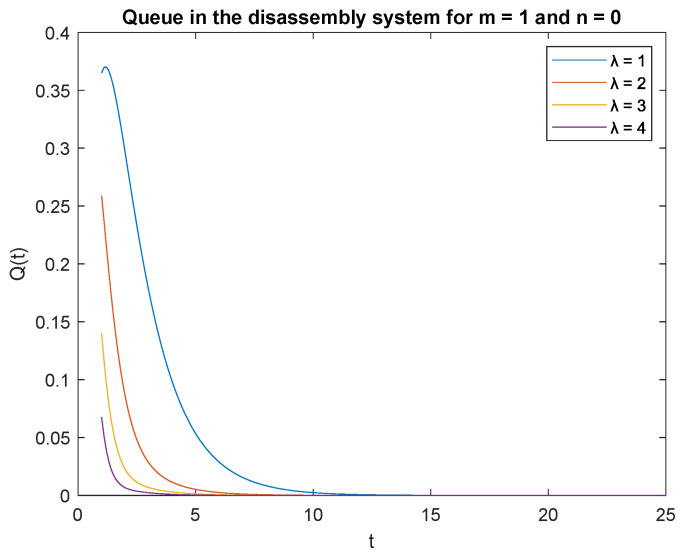
Impact of arrival rate on probability P{X(t)=1|X(0)=0}.

**Figure 11:**
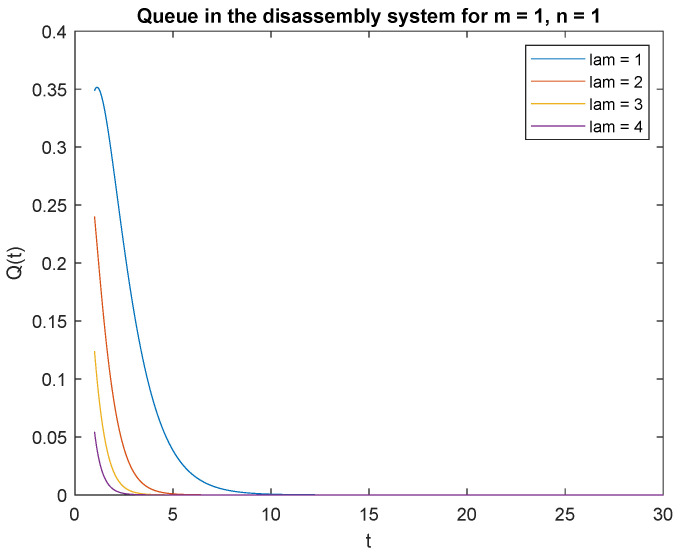
Impact of arrival rate on probability P{X(t)=1|X(0)=1}.

Observe probabilities $P\{X\left(t\right)=1\;|\;X_{0}=0\}$ (Figure 10) and $P\{X\left(t\right)=1\;|\;X_{0}=1\}$ (Figure 11), the transient conditional distributions increase with decreasing inter-arrival rates λ=1, λ=2, λ=3, and λ=4. The transient conditional distributions increase shortly after opening the disassembly system only for inter-arrival rate λ=1. The transient conditional distributions decrease after opening the disassembly system for inter-arrival rates λ=2, λ=3, λ=4 starting from the peak at the beginning of simulation for both probabilities Figure 10 and Figure 11).

In summary, considering the scenarios where the capacity of the accumulating buffer B=1 and the probabilities of m=0 or m=1 and n=0 or n=1, the following conclusions can be drawn:

Inter-arrival rate has a strong influence on the probabilities. The effect of the inter-arrival time is clearly visible and the differences between corresponding probabilities are relatively large.

The transient conditional distributions increase as λ increases.

The transient conditional distributions increase shortly after opening the disassembly system as $\lambda_{i}$ decreases and *m* increases.

### 7.4. Impact of Disassembly Rate

**Figure 12:**
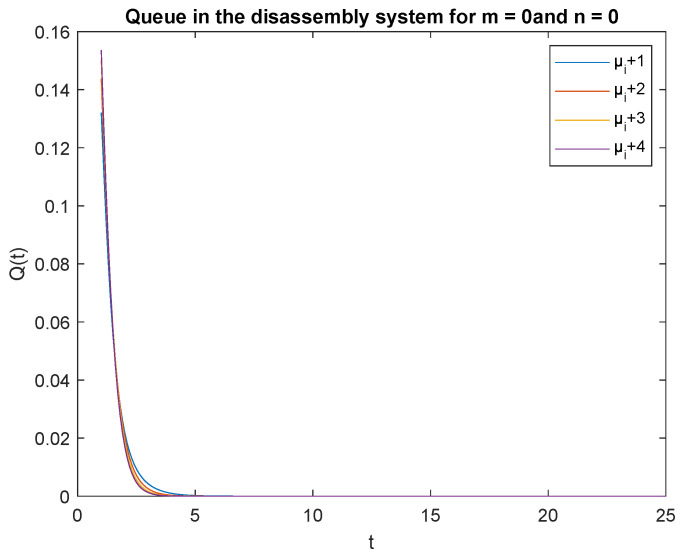
Impact of disassembly rate on probability P{X(t)=m=0|X(0)=n=0} for λ=4.

Now analyze the impact of disassembly rates. Consider three scenarios where the parameters of the CDF describing disassembly times are $\mu_{1}$ = 4, $\mu_{2}$ = 3 and $\mu_{3}$ = 2 and are increased by /1/, 2/, 3/, and 4. The arrival rate is constant λ=4 task/min for the product. The traffic load and capacity of the accumulating buffer are as in the previous subsection. We have the capacity (traffic load) of the system, that is the transient conditional distributions P{X(t)=0|X(0)=0} for increasing disassembly times visualized in Figure 12.

**Figure 13:**
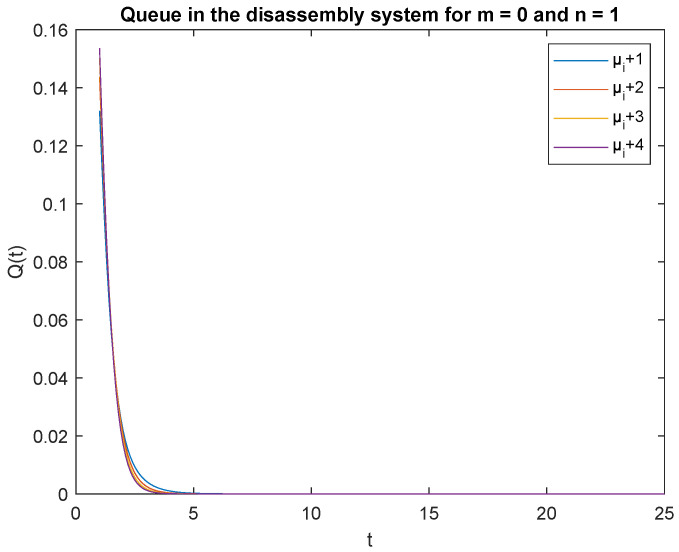
Impact of disassembly rate on probability P{X(t)=m=0|X(0)=n=1} for λ=4.

**Figure 14:**
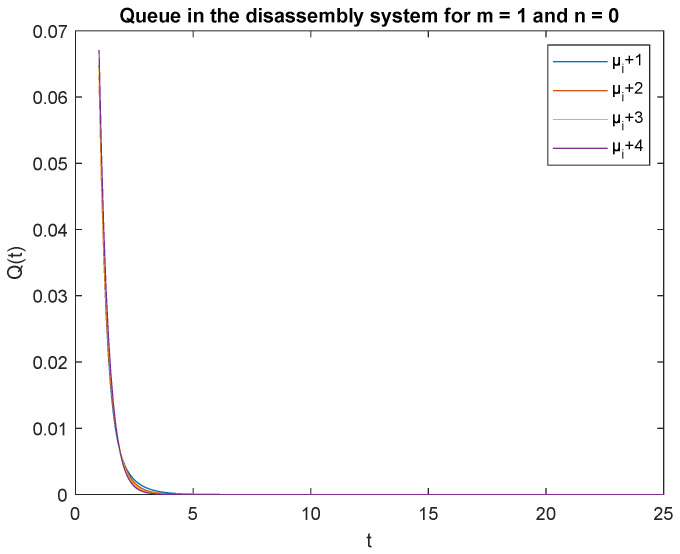
Impact of disassembly rate on probability P{X(t)=1|X(0)=0} for λ=4.

**Figure 15:**
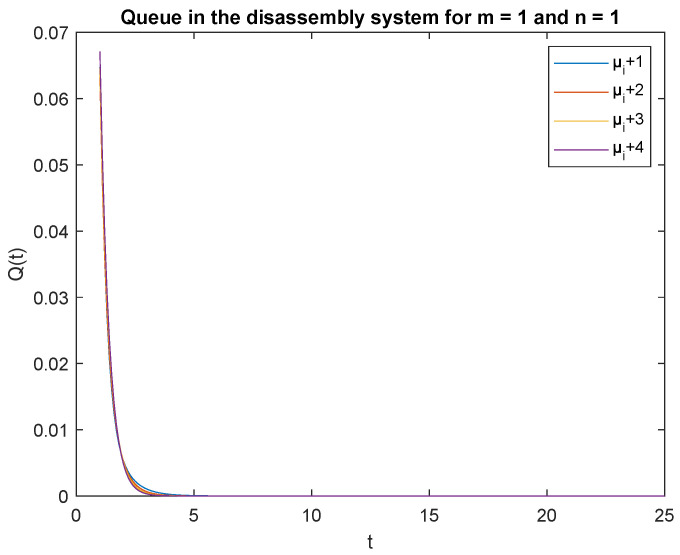
Impact of disassembly rate on probability P{X(t)=1|X(0)=1} for λ=4.

Observe that the probabilities P{X(t)=0|X(0)=0}, P{X(t)=0|X(0)=1} and P{X(t)=1|X(0)=0}, P{X(t)=1|X(0)=1} decrease shortly after opening the disassembly system, down to 5 s (Figure 12, Figure 13, Figure 14 and Figure 15). The probabilities peak just after opening the disassembly system.

Considering the scenarios in the disassembly system where the buffer capacity, B=1 and there is no job or only one job at the beginning, the probabilities of no job m=0 at time *t* is slightly higher than the probabilities of one job m=1.

**Figure 16:**
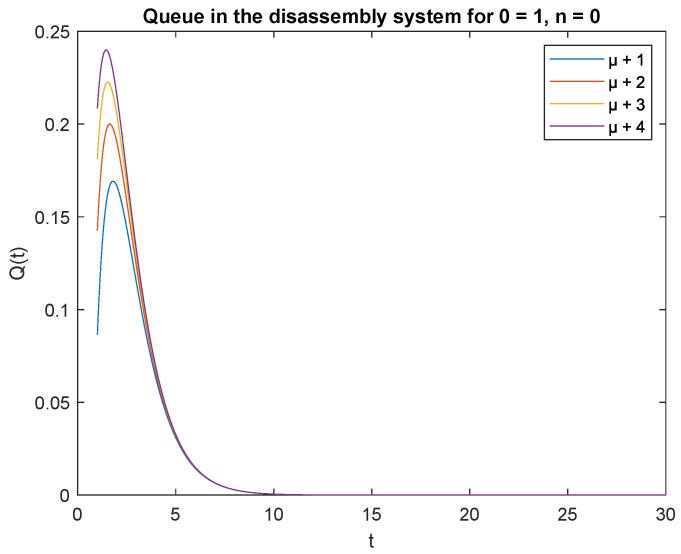
Impact of disassembly rate on probability P{X(t)=m=0|X(0)=n=0} for λ=1.

**Figure 17:**
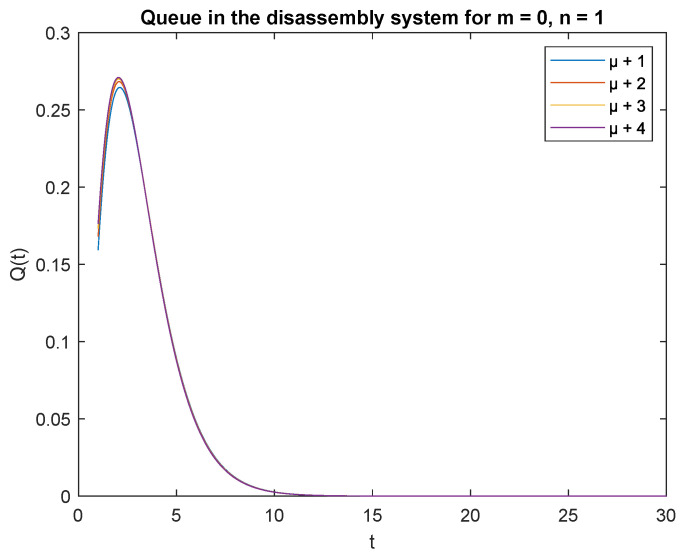
Impact of disassembly rate on probability P{X(t)=m=0|X(0)=n=1} for λ=1.

**Figure 18:**
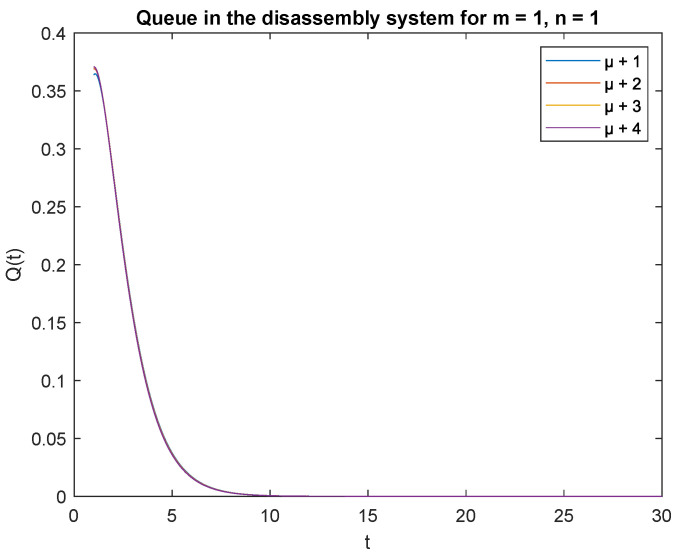
Impact of disassembly rate on probability P{X(t)=1|X(0)=0} for λ=1.

To support the conclusion that the disassembly rate has very little impact on the probabilities, we also simulate the probabilities P{X(t)=0|X(0)=0}, P{X(t)=0|X(0)=1} and P{X(t)=1|X(0)=1} where the arrival intensity, λ=1 task/min and traffic loads $p_{i}=\left\{0.2,0.3,0.5\right\}$ are constant. The conclusion is correct except for the probability P{X(t)=0|X(0)=0} (compare Figure 16, Figure 17 and Figure 18).

The following conclusions are given:

The disassembly rates have little (Figure 16) or very little impact on the probabilities (Figure 12, Figure 13, Figure 14 and Figure 15, Figure 17 and Figure 18).

The four probabilities are highest just after opening the system (Figure 12, Figure 13, Figure 14 and Figure 15), which means that the system starts the disassembly process right after opening. The disassembly system immediately goes into the vacation mode due to the lack of disassembly tasks.

### 7.5. Impact of Traffic Load on a Product with a Given Quality

Finally, let us check the response of the transient queue-size distribution to changes in the traffic load of a product of different quality classified for regeneration, reuse, or recycling. There are three traffic load scenarios: 20%, 30%, and 50% for regeneration, reuse, or recycling, $p_{i}=\left\{0.2,0.6,0.2\right\}$ and $p_{i}=\left\{0.5,0.3,0.2\right\}$, where capacity of the accumulating butter B=1. The parameters of the CDF describing the disassembly time are $\mu_{1}=2$, $\mu_{2}=4$, and $\mu_{3}=6$ for regeneration, reuse, or recycling, respectively. Keeping the arrival intensity constant λ=4 task/min for the product.

**Figure 19:**
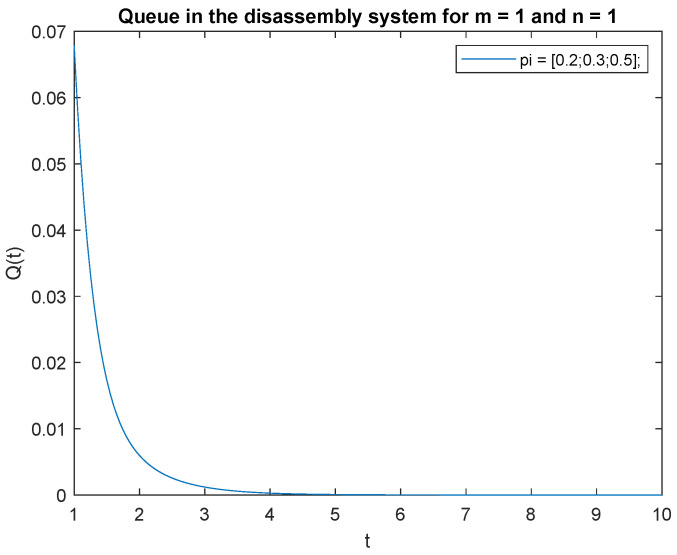
Impact of traffic load described by $p_{i}=\left\{0.2,0.3,0.5\right\}$ on P{X(t)=m=1|X(0)=n=1} for λ=4.

**Figure 20:**
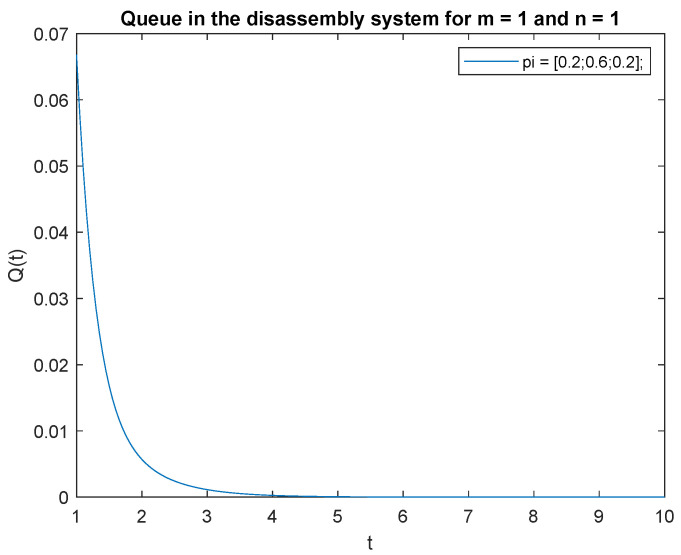
Impact of traffic load described by $p_{i}=\left\{0.2,0.6,0.2\right\}$ on P{X(t)=m=1|X(0)=n=1} for λ=4.

**Figure 21:**
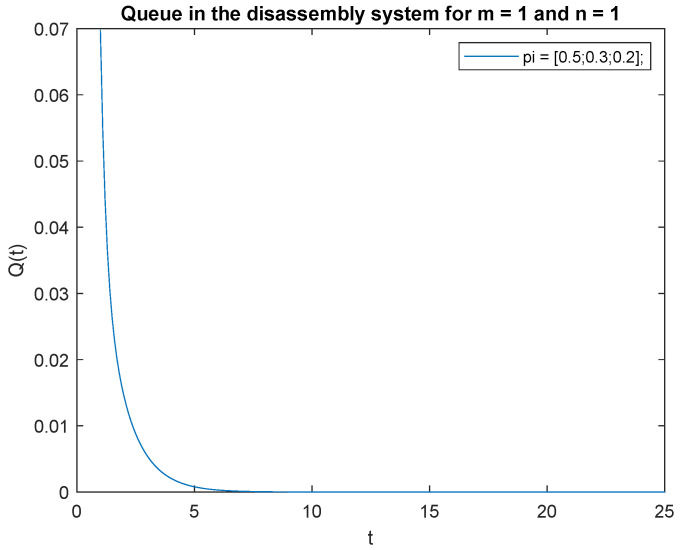
Impact of traffic load described by $p_{i}=\left\{0.5,0.3,0.2\right\}$ on P{X(t)=m=1|X(0)=n=1} for λ=4.

Consider the scenario where the traffic load $p_{i}=\left\{0.2,0.3,0.5\right\}$, the probabilities that of one job, m=1 at time *t* and one job at the beginning, n=1 in the input buffer, **P**{*X*(*t*) = 1|*X*(0) = 1} are presented in Figure 19. The probability is highest right after opening the system, then drops to 0 after about 4 s. A similar phenomenon is observed for the traffic load $p_{i}=\left\{0.2,0.6,0.2\right\}$, the probability of one job, m=1 at time *t* and of one job, n=1 at the beginning in the input buffer (Figure 20). The probabilities of one job, m=1 at time *t* and one job, n=1 at the beginning in the input buffer are higher for the traffic load $p_{i}=\left\{0.5,0.3,0.2\right\}$ (Figure 21) than for the loads presented in Figure 19 and Figure 20.

**Figure 22:**
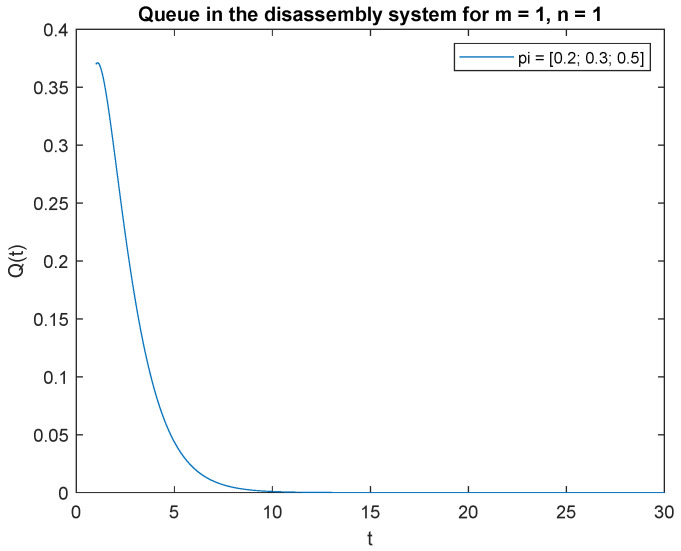
Impact of traffic load described by $p_{i}=\left\{0.2,0.3,0.5\right\}$ on P{X(t)=m=1|X(0)=n=1} for λ=1.

**Figure 23:**
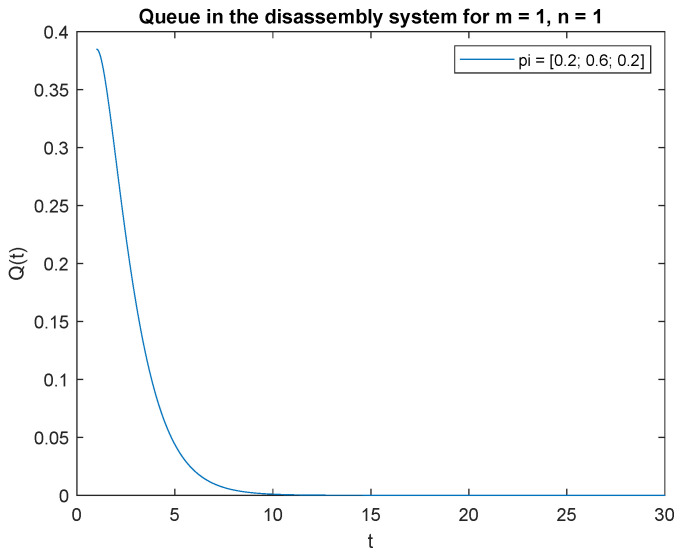
Impact of traffic load described by $p_{i}=\left\{0.2,0.6,0.2\right\}$ on P{X(t)=m=1|X(0)=n=1} for λ=1.

**Figure 24:**
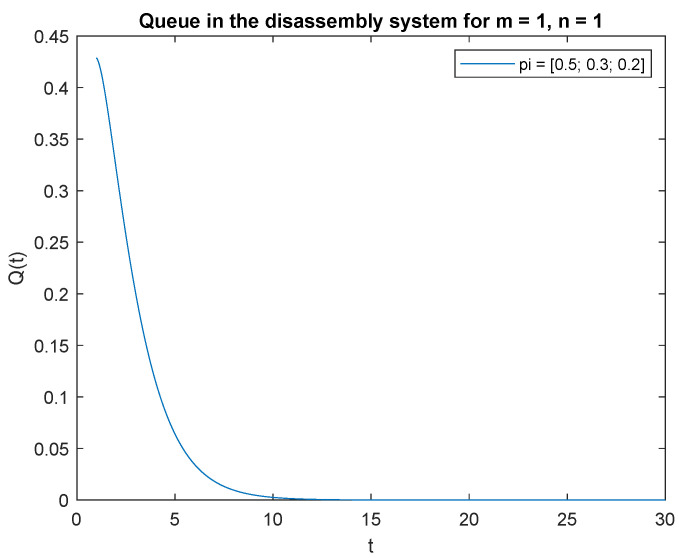
Impact of traffic load described by $p_{i}=\left\{0.5,0.3,0.2\right\}$ on P{X(t)=m=1|X(0)=n=1} for λ=1.

Let us briefly comment on the recent results. The higher the probability of the product classified for regeneration the higher probability P{X(t)=1|X(0)=1}. The higher probabilities of the product for reuse or recycling the lower impact on probability P{X(t)=1|X(0)=1}. The same conclusion can be drawn for the probabilities and constant λ=1 (Figure 22, Figure 23 and Figure 24).

In order to support the conclusion that the disassembly rate has very little impact on the probabilities, observe probability P{X(t)=1|X(0)=1} where the arrival intensity of the product is constant, λ=1 and the traffic load is variable. The conclusion is correct except for the traffic load $p_{i}=\left\{0.5,0.3,0.2\right\}$ (compare Figure 22, Figure 23 and Figure 24).

The disassembly rate has very little impact on the probabilities when the traffic load is light. The greater the proportion of a job with a longer disassembly time, the greater the impact of disassembly time on the probability P{X(t)=1|X(0)=1} (Figure 22, Figure 23 and Figure 24).

## 8. Conclusions

The problem of sequencing a single-product line with three possible cases of a target component was investigated: recycling, reuse, or regeneration (re-manufacturing). Due to the unknown quality, quantity, and frequency of product returns, the method for estimating the time between the arrival of the product with the target component for re-manufacturing, reusing, or recycling received from the reverse logistics network was presented. A system of equations for the time-sensitive queue-size distribution was established by applying the paradigm of the embedded Markov chain and the total probability law. The presented approach estimates the effect of the initial buffer state, initial buffer capacity, product arrival intensity, product disassembly rate, and traffic load on the probability of one job at time *t*. The presented approach computes the transient queue-size distribution for the digital twin of the disassembly system.

Thanks to reliable predictions of the disassembly system occupancy, it is possible to better plan the disassembly tasks along with limited production capacities. Better use of the disassembly system improves performance indicators.

The most important conclusions were drawn:

Given the effect of the initial buffer state on the transient queue-size distribution, the probabilities of one job at time *t* were higher than the probabilities of no job regardless of the initial buffer state.

Taking into account the impact of the arrival rate on the transient queue-size distribution, the following conclusion was given: the arrival rate had a large impact on the probabilities. The effect of the time between arrivals was clearly visible, and the differences between corresponding probabilities were relatively large. The transient conditional distributions increased with the increase in the intensity of arrivals. The transient conditional distributions increased shortly after the opening of the disassembly system for less and less intensity of arrivals and a single job in the input buffer.

The impact of disassembly rates on the transient queue-size distribution was very small at the beginning of our simulations. Finally, we refined our conclusion that the disassembly rates had very little effect on the probabilities when the traffic load is light. The greater the proportion of tasks with longer disassembly times, the greater the impact of disassembly time on the probability of one job at time *t*, where there was one job at the beginning.

The probabilities were highest right after the system was opened, which means that the system started the disassembly process right after the system was opened. The disassembly system immediately went into the vacation mode due to a lack of disassembly tasks.

In the traffic load, the product qualified for regeneration had the greatest impact on the size distribution of the transition queue. 

## Data Availability

Data sharing not applicable.
